# Bibliometric Analysis of Cathepsin B Research From 2011 to 2021

**DOI:** 10.3389/fmed.2022.898455

**Published:** 2022-07-06

**Authors:** Xiaoli Yang, Hua Yin, Deyu Zhang, Lisi Peng, Keliang Li, Fang Cui, Chuanchao Xia, Zhaoshen Li, Haojie Huang

**Affiliations:** ^1^Department of Gastroenterology, General Hospital of Ningxia Medical University, Ningxia Medical University, Yinchuan, China; ^2^Department of Gastroenterology, Changhai Hospital, Navy/Second Military Medical University, Shanghai, China; ^3^Department of Gastroenterology, The First Affiliated Hospital of Zhengzhou University, Zhengzhou, China

**Keywords:** cathepsin B, pancreatic diseases, Research Frontier, bibliometrics, visualization

## Abstract

Cathepsin B (CTSB) is a lysosomal protease implicated in the progression of various diseases. A large number of CTSB-related studies have been conducted to date. However, there is no comprehensive bibliometric analysis on this subject. In our study, we performed quantitative analysis of CTSB-related publications retrieved from the Science Citation Index Expanded (SCIE) of the Web of Science Core Collection (reference period: 2011–2021). A total of 3,062 original articles and reviews were retrieved. The largest number of publications were from USA (*n* = 847, 27.66%). The research output of each country showed positive correlation with gross domestic product (GDP) (*r* = 0.9745, *P* < 0.0001). Active collaborations between countries/regions were also observed. Reinheckel T and Sloane BF were perhaps the most impactful researchers in the research landscape of CTSB. Plos ONE was the most prevalent (119/3,062, 3.89%) and cited journal (3,021 citations). Comprehensive analysis of the top citations, co-citations, and keywords was performed to acquire the theoretical basis and hotspots of CTSB-related research. The main topics included CTSB-related cancers and inflammatory diseases, CTSB-associated cell death pattern, and the applications of CTSB. These results provide comprehensive insights into the current status of global CTSB-related research especially in pancreas, which is worthy of continued follow-up by practitioners and clinicians in this field.

## Introduction

Cathepsin B (EC 3.4.22.1, CTSB), a lysosomal cysteine protease in the Papain family, exhibits both endopeptidase and exopeptidase activity. It can act both extracellularly and as an activator of trypsinogen within the cell (1–3). CTSB is believed to colocalize with trypsinogen in the lysosomes, and subsequently activate the trypsinogen causing acute pancreatitis (4). In a study by Sendler et al., trypsinogen was shown to be activated by endocytosed CTSB in macrophages, promoting pancreatitis in mice (5). Additionally, CTSB may play a role in tumor initiation, proliferation, angiogenesis, and metastasis, and promote carcinogenesis in pancreatic tissue (6, 7).

*CTSB* gene is located at chromosome 8p22 and contains 13 exons and 11 introns (8, 9). Pre-mature CTSB protein is a 44 kD zymogen, and an intermediate formation is 33 kD with a single chain. The active CTSB protein is formed after maturation processing and has a 27–29 kD heavy chain and a 4–6 kD light chain (8, 10, 11). In 1991, the crystal structure of human CTSB in its two-chain form with 2.15 Å resolution was identified, and it was the first determined crystal structure of human cathepsin (12). It is roughly disc-shaped and 50 Å in diameter with 30 Å thickness and a distinct active site cleft. The polypeptide chain folds into two distinct domains interacting through an extended polar interface that opens to V-shaped active site cleft, similar to the related cysteine protease papain, actinidin, and calotropin D (12). CTSB can act as a peptidyl dipeptidase in neutral pH, which as an exopeptidase removes dipeptides from the C-terminus of proteins and peptides; CTSB can also act as an endopeptidase with relatively broad specificity with slight preference for basic residues in acidic pH to cleave internal peptide bonds (12). The dual activation relies on the occluding loop (the covalently closed circular region between Cys108 and Cys119 residues) which is a unique structural element of CTSB (13). The loop partially blocks the end of the active-site cleft and positions a positively-charged imidazole group of a histidine residue to accept the negative charge at the C-terminus of the substrate, enabling CTSB to act as an exopeptidase; in addition, it has a flexible structure that can adopt a conformation that no longer blocks the binding cleft, allowing the enzyme to act as an endopeptidase (14). However, CTSB is a less effective endopeptidase than some other members of the papain family, probably because of the energy cost of altering the conformation of the occluding loop (15). CTSB locates in the islet endocrine cells and acinar cells in pancreas (16, 17). Changes in the expression and distribution of CTSB is associated with various disease. In the study by Saluja et al., redistribution of CTSB in the acinar cells of pancreas and colocalization with trypsinogen led to the development of acute pancreatitis (16). Moreover, the vesicles staining for CTSB were observed more peripheral in the aggregated cells and the redistribution of CTSB vesicles toward the cell periphery may be induced by the acidic pericellular pH, which facilitates the progression and development of pancreatic cancer (18). However, owing to the complexity of etiology and pathogenesis of CTSB-related diseases, especially the enormous interactions of different signal pathways, further studies are required to unravel the exact role of CTSB especially in pancreas.

Bibliometric analysis is one of the most extensively used approaches for assessing the quantity, quality, reliability, and influence of the existing research achievements. It can provide comprehensive integration, interpretation, and analysis of the evolution and dynamics of scientific information in a specific field. The present bibliometric study encompassed 3,062 publications in the field of CTSB published during the past decade to obtain a global understanding of CTSB-related research. The key aspects analyzed included annual output, most productive countries/regions and their gross domestic product (GDP), the top scientific journals, the number of citations and co-citations, co-occurrence and burst detection of keywords. The potential research hotspots and latest trends identified in this study may provide a valuable reference for researchers interested in the CTSB field.

## Methods

### Data Source

The Clarivate Analytics SCIE database was searched on October 1, 2020, to obtain CTSB-related publications in the latest decade (2011–2021) with no language preference. The retrieval strategy was as follows: ALL FIELDS: (“cathepsin B”) OR ALL FIELDS: (“CTSB”) AND ALL FIELDS: (pancrea^*^) Refined by: DOCUMENT TYPES: (ARTICLE OR REVIEW) Timespan: 2011–2021. Indexes: SCI-EXPANDED. The CTSB-relativity screen was performed by three authors independently using the EndNote X9, and the differences were discussed until consensus. Then the CTSB-relativity studies were screened from the CTSB-relativity data with the limitation of keywords to “Pancreatic Cancer”, “Cancer of Pancreas”, “Pancreas Neoplasm” or “pancreatitis”. All targeted data were acquired in text format on October 1, 2020, and formatted into Prism software (version 8.3.0). The following bibliometrics and visualization tools VOSviewer (1.6.17) and CiteSpace (5.8.R2) were used for analysis.

### Statistical Analysis

Prism software (version 8.3.0) was used to conduct the histogram, the correlation analysis of top 20 productive countries/regions and their GDPs (deadline: October 2021). The collaborations of countries/regions or authors, top citation journals and key references were visualized by VOSviewer. VOSviewer plots show different bubbles representing elements such as authors, journals, references, and keywords. The area of the bubbles represents the frequency of target elements. The line and its thickness reflect the collaborations and the strength of the relationship between two bubbles (19, 20). In the present study, settings of the VOSviewer were: full counting with threshold (T) based on corresponding-elements. The Biblioshiny program of R-bibliometrix was utilized to identify the most impactful of authors.

## Results

### Annual Output

**Figure 1:**
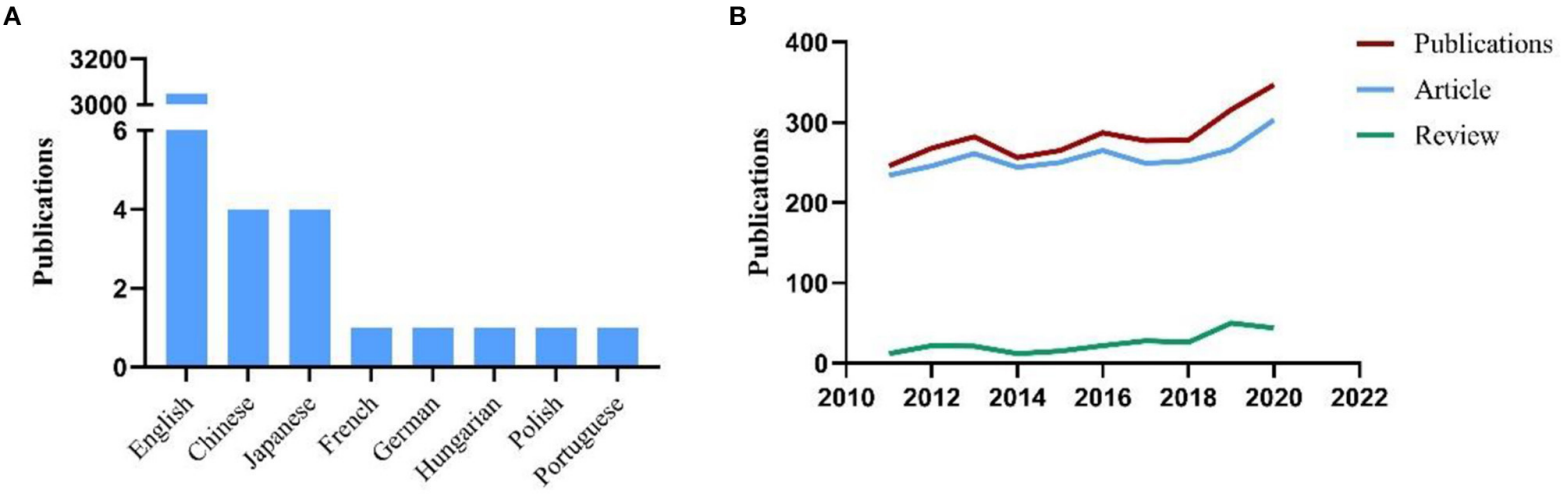
**(A)** The languages of publications in CTSB field; **(B)** Number of publications by year (2011–2020).

A total of 3062 publications related to CTSB in the study reference period were retrieved from the SCIE database. The data used for the subsequent analysis were obtained from the search results of this database. “Original research articles” and “review articles” accounted for 2,784 (90.92%) and 278 (9.08%) publications, respectively. English was the predominant language of publication in this field, accounting for 94.68% (3,049/3,062) of all publications. The other languages were Chinese (4/3,062, 0.13%), Japanese (4, 0.13%), and French, German, Hungarian, Polish and Portuguese (one article each) (Figure 1A). Figure 1B shows the annual output of publications in the field of CTSB; a relatively increasing trend was observed in the study reference period.

### Country/Region

**Figure 2:**
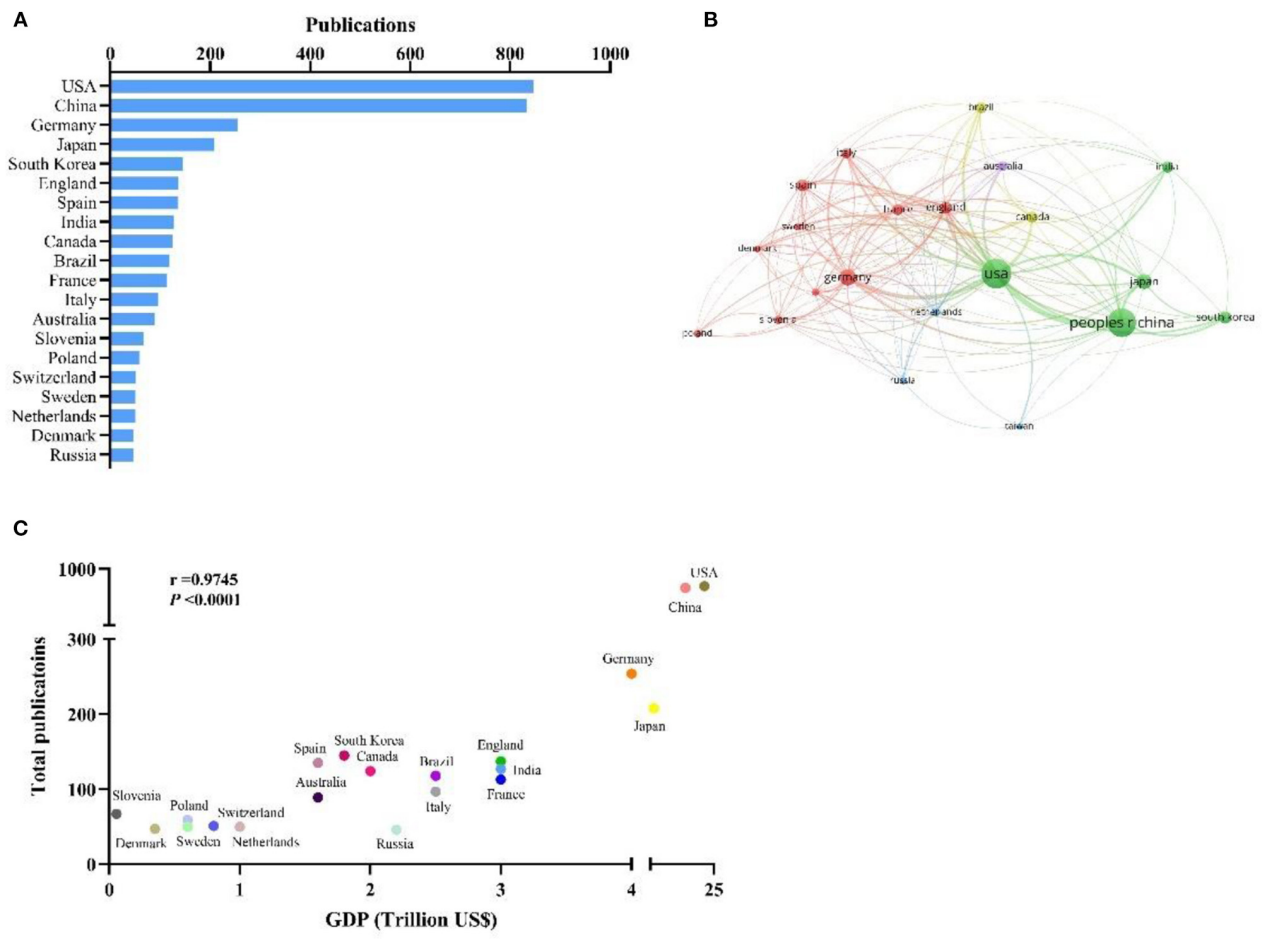
**(A)** The top 20 most productive countries for CTSB research; **(B)** Network map showing distribution of collaboration between Countries/Regions; **(C)** Correlation between total articles published and GDP of 15 highest-output countries.

The contribution of various countries/regions was estimated by the number of publications. The top 20 productive countries/regions acquired from SCIE database are shown in Figure 2A. USA and China accounted for the highest number of papers (*n* = 847 and 833, respectively), followed by Germany (*n* = 254), Japan (*n* = 208), South Korea (*n* = 145), England (*n* = 137), Spain (135), India (*n* = 127), Canada (*n* = 124), Brazil (*n* = 118), and France (*n* = 113). The network map (T = 46) shows the collaboration between these top countries/regions (Figure 2B). The size of the bubbles represents the number of publications of each country/region. The colors represent different clusters, the cluster represents the similar research topic in the CTSB field. The line and its thickness reflect the collaboration and the strength of relationship between two countries/regions. Active collaborations were seen among various countries; for instance, USA cooperated closely with China, Germany, England, Japan, Australia, South Korea and India. Moreover, the relationship between research output and economic development was evaluated for the 20 most productive countries using GraphPad Prism; the GDP data of countries were retrieved from the World Bank Open Data [October 1, 2021(21)]. The results showed a distinct positive relationship between research output and GDP of the top 20 countries (r = 0.9745, *P* < 0.0001; Figure 2C).

### Authors

**Figure 3:**
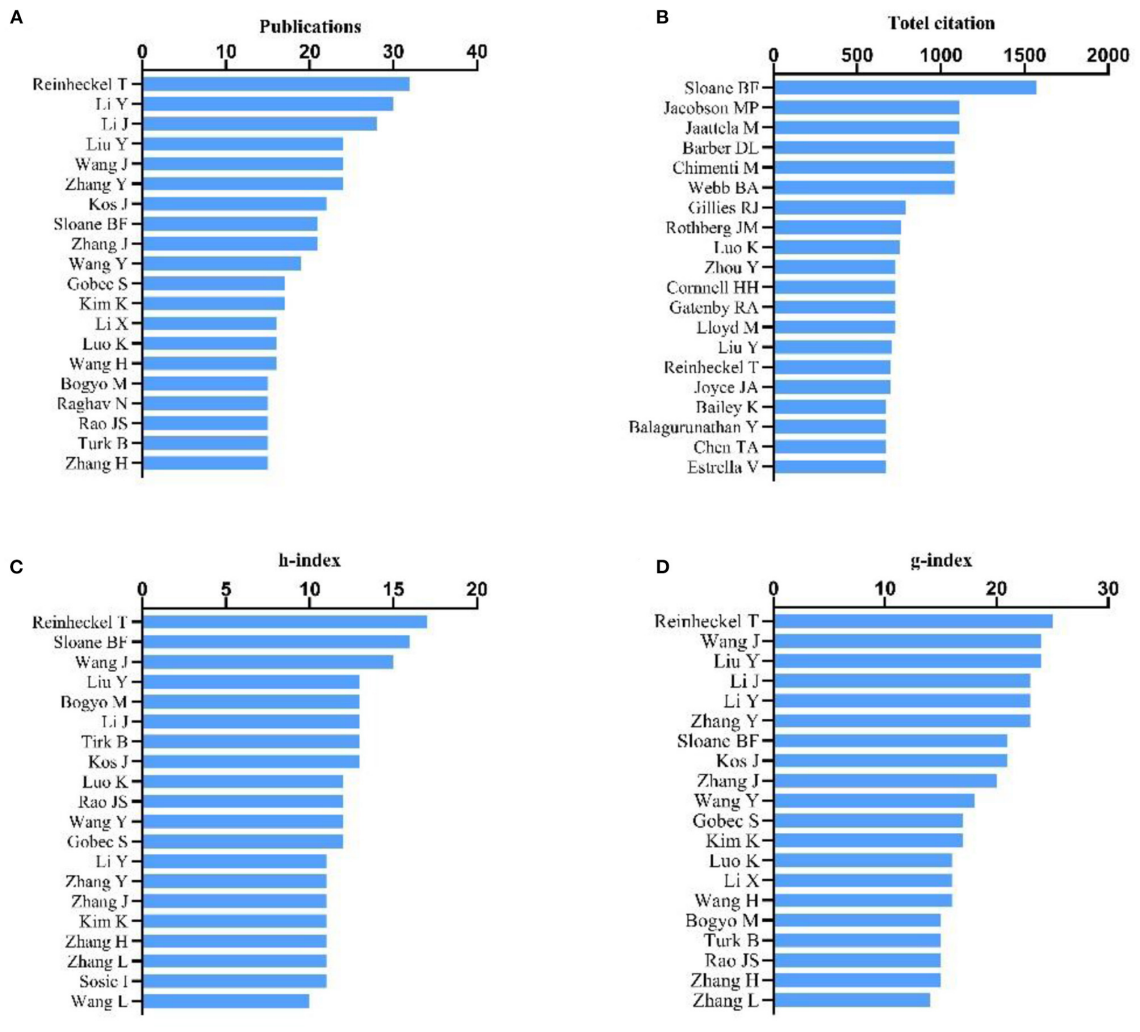
Analysis of authors. **(A)** Number of publications from different authors; **(B)** Total citations in the research field from different authors; **(C)** h-index of publications from different authors; **(D)** g-index of publications from different authors.

The influential authors were comprehensively appraised based on four parameters: the total number of their publications, the total citation count, h-index, and g-index. The influence of scientists and their papers was evaluated by citation count. The h-index, which is a well-known metric to determine the quality of a scientist, is calculated by the citation count of papers. The g-index was introduced as an improvement of the h-index (22, 23). Reinheckel T was found to be the most prolific author with a total of 32 published articles (32/3,062, 1.05%), followed by Li Y (30, 0.98%) and Li Y (28, 0.91%) (Figure 3A). According to the number of citations in this field, Sloane BF ranked first (1,572 citations), while Jacobson MP (1,114 citations) and Jaattela M (1,109 citations) ranked second and third, respectively. Barber DL, Chimenti M, and Webb BA, coauthors of the most cited article, showed equal contributions with 1,080 citations each (Figure 3B). Publications by Reinheckel T had the highest h-index (17), followed by those of Sloane BF (16), Wang J (15), Liu Y (13), Bogyo M (13), Li J (13), Turk B (13), and Kos J (13) (Figure 3C). The g-index of publications by Reinheckel T (24) also ranked first, followed by those from Wang J and Liu Y (24, each), then Li J, Li Y and Zhang Y (23 each) (Figure 3D).

### Journals

**Figure 4:**
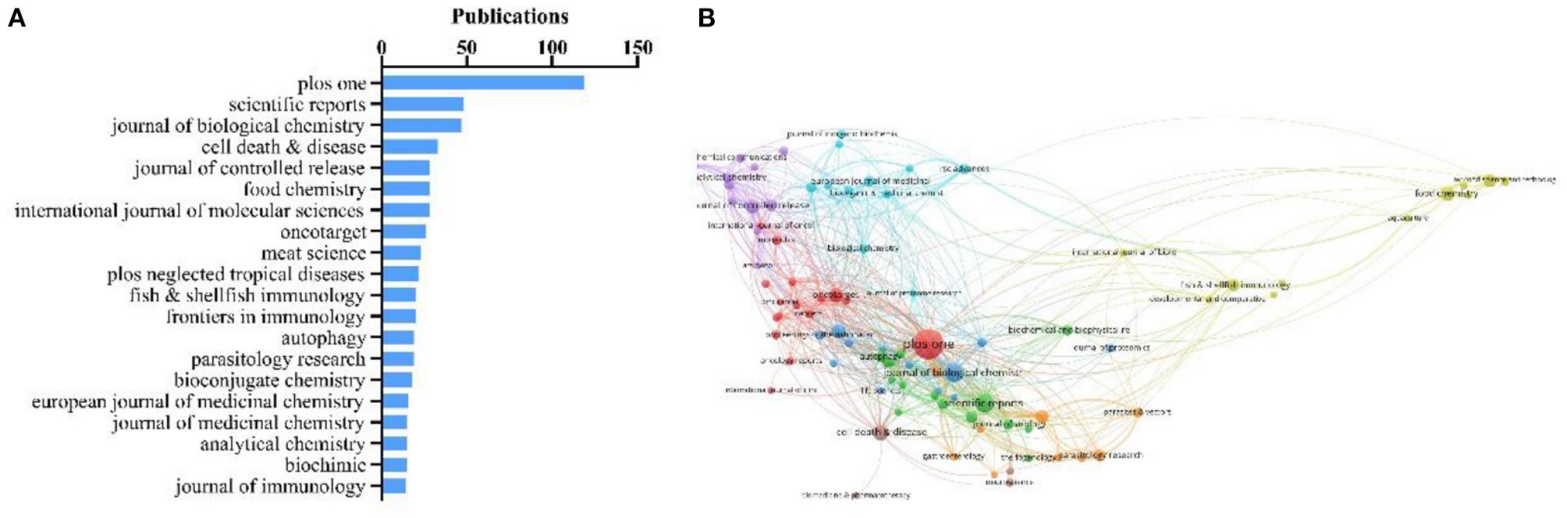
**(A)** Top 20 journals assessed by number of publications; **(B)** Network map of scholarly journals (T = 7) (produced by citation).

Academic journals publishing CTSB-related research were evaluated by total publications. The top 20 journals for CTSB publications ranked by VOSviewer are presented in Figure 4A. Four journals have published more than 30 papers in this field, of which Plos ONE was way ahead of other journals with 119 publications (119/3,062, 3.89%), followed by Scientific Reports (*n* = 48, 1.57%), Journal of Biological Chemistry (*n* = 47, 1.53%), and Cell Death & Disease (*n* = 33, 1.08%). To visualize the relationships of journals, the citation network map was constructed using journals with ≥7 publications (T = 7) (90/1,068, 8.43%) (Figure 4B). The size of the bubble represents the number of citations per journal. Different colors represent different clusters, the hallmarking of different research topics on the CTSB theme. The line and its thickness reflect the collaboration and the intensity of the mutual citation between two academic journals. As can be seen, Plos ONE, Journal of Biological Chemistry, Cancer Research, Journal of Immunology, ACS Nano, Cell Death & Disease, and Journal of Controlled Release had larger-sized bubbles representing higher journal citations.

### Assessment of Top Literatures

The top citations of publications were evaluated by VOSviewer. Table 1 displays the top eight papers with the highest citations (T = 310). There were 1,080 citations for “Dysregulated pH: a perfect storm for cancer progression” from Nature Reviews Cancer (25), followed by “Acidity Generated by the Tumor Microenvironment Drives Local Invasion” from Cancer Research (26), with 669 citations. The third most-frequently cited article was “Molecular mechanisms regulating NLRP3 inflammasome activation” (24), with 545 citations. The top 8 references with over 100 co-citations are listed in Table 2. The reference with the highest co-citation (*n* = 205) was from Mohamed MM (32), titled “Cysteine cathepsins: multifunctional enzymes in cancer”, a review that exhaustively described the role of cysteine cathepsins in tumor growth, migration, invasion, angiogenesis and metastasis. This was followed by the reviews from Turk V (33) (*n* = 155), Gondi CS (35) (*n* = 113), Barrett AJ (36) (*n* = 112), Boya P (37) (*n* = 109) and Aggarwal N (38) (*n* = 100). The two articles with high co-citations are from Hornung V (34) and Musil D (12). Five of eight top co-cited references are all reviews, which indicates that reviews may be more highly cited than research articles in the co-citation of references. However, these publications no matter reviews or articles encompassed a wide spectrum of CTSB-related research including the properties of cysteine proteases, the molecular mechanisms, the protease related diseases, and biochemical applications.

**Table 1:** Top eight citation analysis of publications on CTSB research.

**Rank**	**References**	**Title**	**Source**	**Country**	**Citations**
1	(25)	Dysregulated pH: a perfect storm for cancer progression	Nature Reviews Cancer	USA	1,080
2	(26)	Acidity Generated by the Tumor Microenvironment Drives Local Invasion	Cancer Research	USA	669
3	(24)	Molecular mechanisms regulating NLRP3 inflammasome activation	Cellular and Molecular Immunology	South Korea	545
4	(27)	Microtubule-driven spatial arrangement of mitochondria promotes activation of the NLRP3 inflammasome	Nature Immunology	Japan	457
5	(28)	Chemotherapy-triggered cathepsin B release in myeloid-derived suppressor cells activates the Nlrp3 inflammasome and promotes tumor growth	Nature medicine	France	435
6	(29)	Specific Light-Up Bioprobe with Aggregation-Induced Emission and Activatable Photoactivity for the Targeted and Image-Guided Photodynamic Ablation of Cancer Cells	Angewandte Chemie International Edition in English	Singapore	346
7	(30)	Proteolytic networks in cancer	Trends in Cell Biology	USA	336
8	(31)	Natively Inhibited Trypanosoma brucei Cathepsin B Structure Determined by Using an X-ray Laser	Science	Germany	314

**Table 2:** Top eight co-citation analysis of references on CTSB research.

**Rank**	**References**	**Title**	**Source**	**Country**	**Co-citations**
1	(32)	Cysteine cathepsins: multifunctional enzymes in cancer	Nature Reviews Cancer	USA	205
2	(33)	Cysteine cathepsins: from structure, function and regulation to new frontiers	Biochimica ET Biophysica ACTA-Proteins and Proteomics	Slovenia	155
3	(34)	Silica crystals and aluminum salts activate the NALP3 inflammasome through phagosomal destabilization	Nature Immunology	USA	116
4	(35)	Cathepsin B as a cancer target	Expert Opinion on Therapeutic Targets	USA	113
5	(36)	Cathepsin-B, cathepsin-H, and cathepsin-L	Methods in Enzymology	England	112
6	(37)	Lysosomal membrane permeabilization in cell death	Oncogene	Spain	109
7	(12)	The refined 2.15 Å X-Ray crystal structure of human Liver cathepsin B: the structural basis for its specificity protein	EMBO Journal	Germany	103
8	(38)	Cathepsin B: multiple roles in cancer	Proteomics Clinical Applications	USA	100

### Keywords Analysis

**Figure 5:**
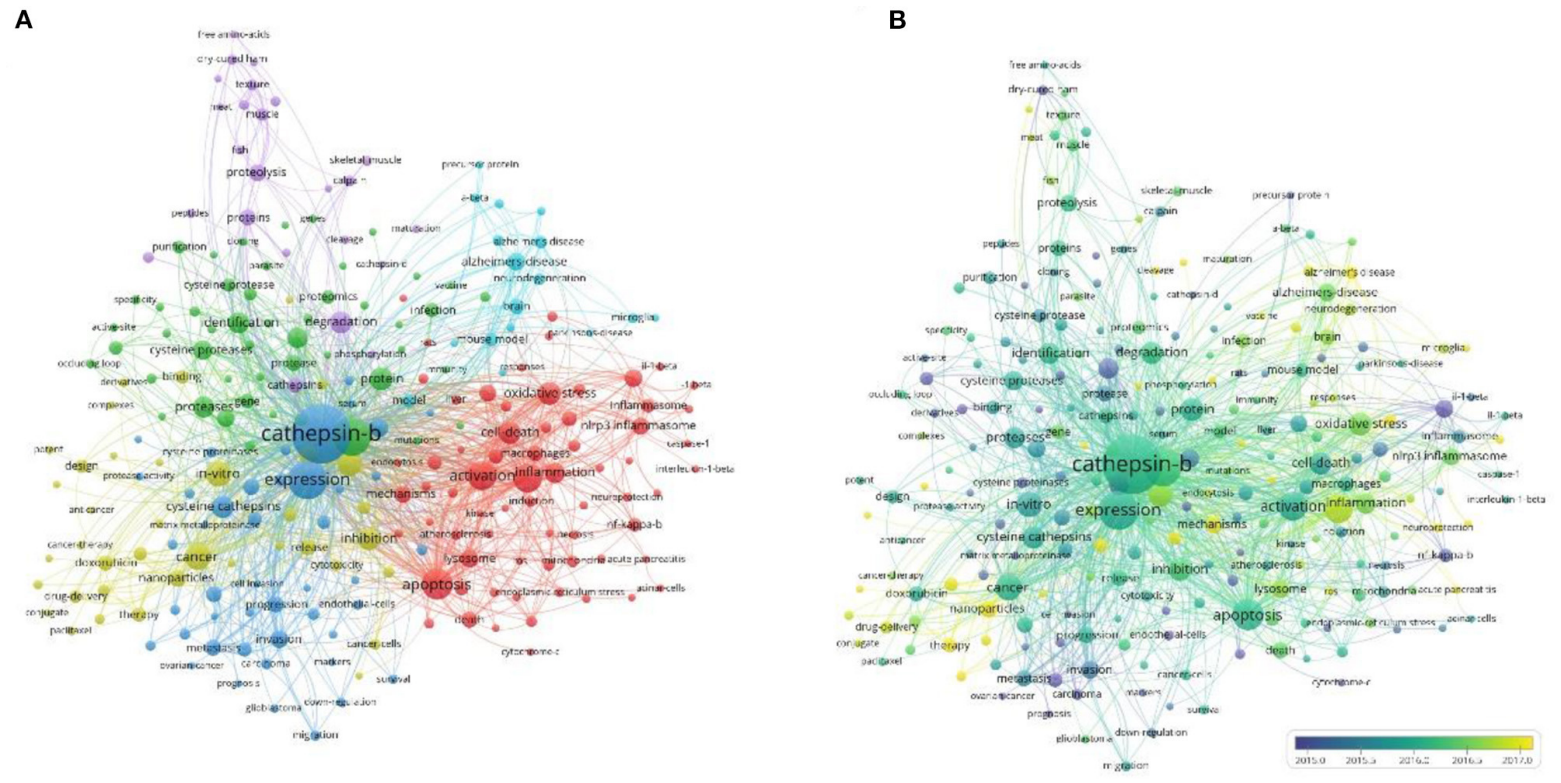
Co-occurrence analysis of keywords. **(A)** Network visualization map of keywords; **(B)** Overlay visualization map of keywords.

**Figure 6:**
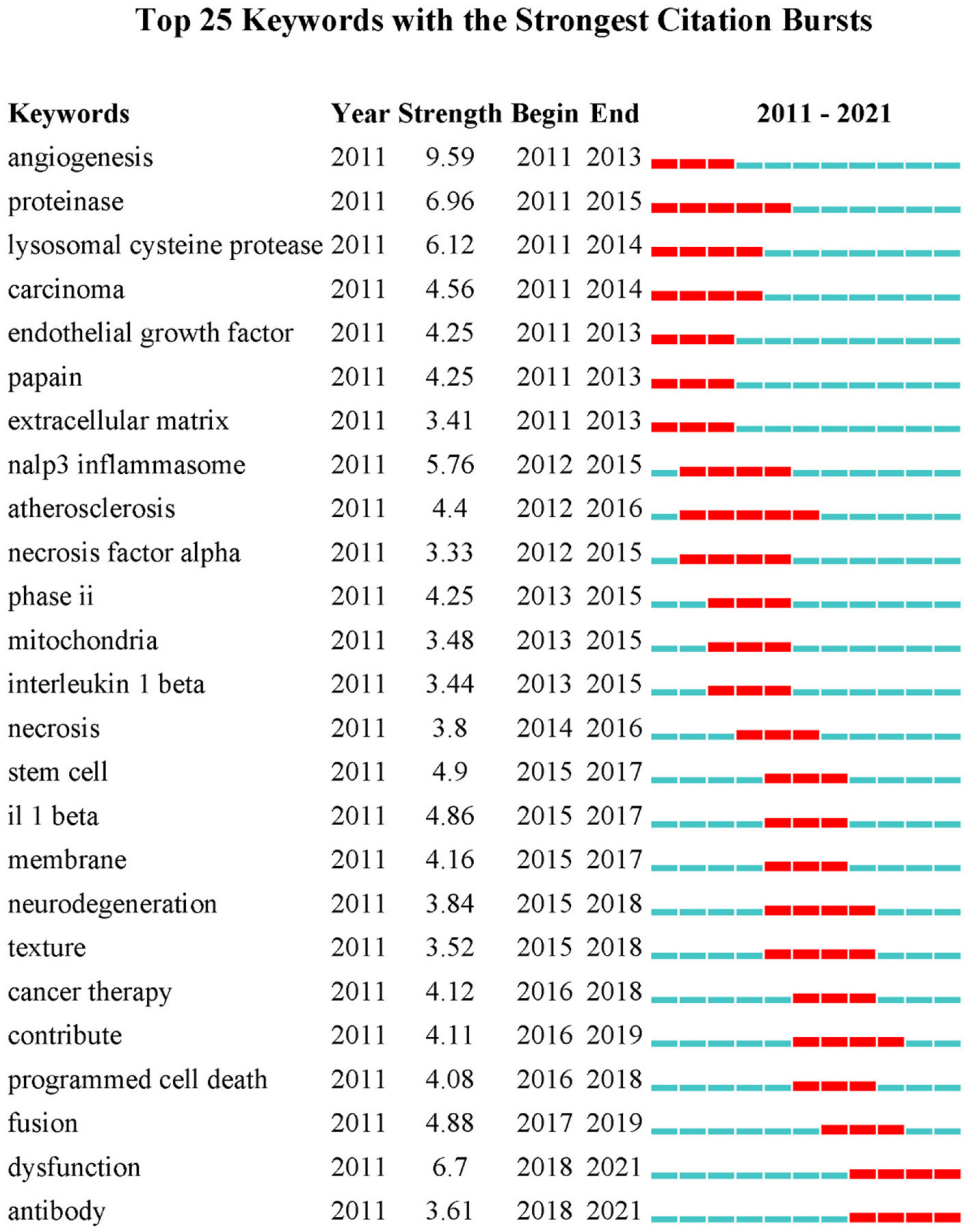
Keywords with the strongest currently ongoing citation bursts (sorted in descending order of Begin).

Co-occurrence relationship is formed between two keywords that appear in the same publication. Strong co-occurrence relationship of all keywords can more accurately reveal research hotspots than a single keyword. A total of 241 keywords were confirmed as occurring more than 20 times with the full counting method; the visualization map is shown in Figure 5. In the network map, the line is a symbol connecting two keywords. The size of bubbles indicates the number of occurrences, and the color represent keyword clustering (Figure 5A). While, the colors presented in the overlay visualization map indicate the average publication year of the identified keywords (Figure 5B). Three broad categories synthesized from six different clusters showed the protease properties and functions, the CTSB-related diseases, and the applications. Furthermore, keyword burst analysis by CiteSpace was performed based on achievements published since 2011 to 2021 (Figure 6). The blue line indicates the time interval, the red line segment in the blue line indicates that a subject was found to have a burst. As shown in Figure 6, during the period from 2011 to 2021, angiogenesis had the highest burst strength (9.59), followed by proteinase (6.96), and dysfunction (6.7). Angiogenesis had a period of burst between 2011 and 2013, while dysfunction had a period of burst between 2018 and 2021. Of note, both dysfunction and antibody had strong bursts starting in 2018.

## Discussion

Bibliometric analysis of the annual output of publications enabled us to characterize the global developments in the field of CTSB. Based on Science Citation Index Expanded database of Web of Science, we performed a bibliometric analysis to characterize the trends in CTSB-related research from 2011 to 2021. We observed a relatively increasing trend of the annual output of publications in CTSB research over the past few years and found that CTSB is still a research hotspot. A vast majority of these studies were published in English, which demonstrated that English is the most popular international language of science and medicine. USA, China, Germany, and Japan were the top four productive countries in the field of CTSB research, indicating their greater contribution to CTSB field. Among the top 20 productive countries, USA and China were way ahead of other countries with more than 800 publications, demonstrating that these two countries may be the potential hubs for research and development in the field of CTSB. Collaborations among the top contributing countries further showed that the CTSB research was a worldwide activity. This kind of cooperation among countries/regions may foster academic sharing and knowledge integration, attract more distinguished scientists to participate in this domain, and accelerate CTSB-related research. Further correlation analysis between GDP and publications showed a tendency of high-GDP countries to gain higher outputs, which demonstrates that these countries pay more attention to scientific research investment, and have sufficient funds to perform advanced research and build advanced research platforms. These elements may accelerate innovation and development of techniques of CTSB research in these countries.

Author analysis helps practitioners to identify the researchers who have the greatest contribution in a particular research field as well as their research level and academic status in this field (39). Analysis of the top 20 most prolific authors was used to screen out active and impactful researchers in this theme. Reinheckel T contributed the largest number of papers with the highest h-index and g-index; Sloane BF had the most citations per paper. These data provide us with the most academically influential and authoritative authors in CTSB field. Research by Reinheckel T was more focused on the CTSB-related diseases, especially pancreatic diseases such as acute pancreatitis and pancreatic ductal adenocarcinoma (2, 40). However, Sloane BF was more focused on the tumors and molecular mechanisms of involvement of CTSB (38, 41, 42). Both Reinheckel T and Sloane BF were the most impactful authors in the CTSB field over the recent decade. Reviewing and analyzing the work of these outstanding academic leaders before starting a new CTSB research would facilitate a better understanding of the basic information in this field. Additionally, analysis of the main scientific journals and their collaborations showed the most prevalent journals and their connections with each other in the CTSB field. This may provide a valuable reference for beginners in this field and make it easier to find the related documents and submit novel discoveries in this field.

The number of citations of a study indicates the relevance and importance of a study in the specific field, and the number of co-cited references exhibits the frequency of two publications being cited together by other publications (19). In our current study, we filtered the publications and references with citations and co-citations from 2011 to 2021 by using the VOSviewer. By combining the top cited publications, co-citation references, and the subsequent analysis of the keywords using network map as well as burst detection, we summarized the basic information and obtained the hotspots of CTSB research field, including the CTSB-related cancer and inflammatory diseases, CTSB-associated cell death pattern, and the applications of CTSB.

Since the first identification of the crystal structure of human CTSB, a multitude of three-dimensional structures of CTSB and their compounds have been identified. All the studies about the structure of CTSB have provided basic information for researchers to design specific targeting inhibitors and develop safe drugs for treatment of CTSB-related diseases. The role of CTSB in carcinogenesis was first identified decades ago (43). It was shown to play an important part in the process of pancreatic carcinogenesis, including cancer-related angiogenesis, malignant invasion, and cancer metastasis (30, 33, 38, 40). Researchers have identified high expression of CTSB in human pancreatic ductal adenocarcinomas and pancreatic cancer stem cells, and demonstrated its association with poor survival and surgical outcomes (44, 45). CTSB exhibits optimal activity in a slightly acidic pH (46, 47). Webb BA and Estrella V identified that acidic pH in the tumor microenvironment may drive malignant transformation and cancer progression (25, 26). The potential underlying mechanism is that pericellular pH leads to the redistribution of CTSB to the surface of malignant cells and accelerates the secretion of active CTSB, which may facilitate invasive growth of these cells (18). CTSB can degrade the extracellular matrix, such as collagen, matrix fiber and proteoglycans, to cause dissolution of the tumor matrix and the basement membrane (48–50), promoting invasion and metastasis. Kim verified overexpression of CTSB in tumor samples and identified its association with increased risk of lymph node metastasis (51). Additionally, CTSB is a well-known candidate activator of trypsinogen in pancreatic acinar cells (52).

Sendler M and Fortunato F also found an association between high expression of CTSB and increased severity of pancreatitis (5, 53). However, enteropeptidase (enterokinase), which is the known physiological activator of trypsinogen and has high catalytic activity for trypsinogen, is involved in the progression of acute pancreatitis (54, 55). To date, there is inadequate evidence to suggest that the role of CTSB precedes that of enteropeptidase or that there is an interaction between the two in the occurrence and development of acute pancreatitis. This may be worthy of future research. Nonetheless, inhibiting the overexpression of CTSB and maintaining its dynamic balance in organism may help control or alleviate CTSB-related cancer and inflammatory diseases.

Studies have identified the underlying biological mechanism by which CTSB promotes programmed cell death (56–59). The release of active CTSB from the damaged lysosomes induces mitochondrial dysfunction which increases cytosol-induced release of cytochrome c from mitochondria resulting in activation of caspase and cell apoptosis (37, 57, 60). Release of reactive oxygen species from mitochondria may also cause lysosomal membrane injury and CTSB leakage from lysosome into cytoplasm contributing to the occurrence of apoptotic cell death (61, 62). However, owing to its pH-dependent bidirectional interactions, CTSB plays myriad roles. It serves as a pro-oncogenic molecule involved in ECM degradation, angiogenesis and metastatic induction; as an anti-apoptotic molecule suppressing the TLR3 pathway and maintaining a pro-survival state; and as a pro-apoptotic molecule involved in autophagy and immune response (35). Additionally, Wang et al. observed that CTSB released from lysosomes promoted severe acute pancreatitis by activating NLRP3 inflammasome which induced self-cleavage of caspase-1 and its maturation into an activated form; this in turn promoted the maturation and secretion of various proinflammatory cytokines including IL-1β and IL-18, triggering caspase-1-induced pyroptosis (3). Pyroptosis is a newly-identified pathway of programmed cell death, characterized by rapid lytic cell death through caspase-1 activity. Recent studies have revealed other caspases (caspase-11 and its human orthologs caspase-4 and caspase-5, and the apoptotic effector caspase-3) which cleave gasdermins (GSDMs) to trigger pyroptosis and induce altered levels of host metabolites and environmental irritants (56, 63–66). The activated CTSB binds to NLRP3 inflammasome to activate caspase, resulting in the expression of GSDMD-N that oligomerize within the plasma membrane to form pores. Excessive pore formation compromises the integrity of the plasma membrane, causing a lytic form of cell death known as pyroptosis. A recent study has identified that CTSB as an autophagy regulator plays an active role in the degradation of autophagic substrate engaged in ferroptosis (a type of iron-dependent oxidative cell death driven by lipid peroxidation) (67). In addition to apoptosis and pyroptosis, other cell death patterns including necroptosis and alkaliptosis (a pH-dependent form of regulated cell death) have been shown to be caspase-dependent and to participate in the progression and development of pancreatic cancer (68). Whether CTSB plays a key role in these modes of cell death is not clear, especially in the context of pancreatic diseases. Considering the complex nature of interaction among proteases, both upstream and downstream pathways of CTSB may affect each other. Thus, much headway needs to be made before the specific mechanism of participation of CTSB can be unraveled.

Owing to the aberrant expression of CTSB in disease conditions, it has been used as a therapeutic target. Wang et al. integrated fibronectin-targeting MR imaging and CTSB-activatable fluorescence imaging for accurate diagnosis and further selective therapy of cancer (69). A drug delivery system targeting the extracellular CTSB through a highly selective CTSB inhibitor (NS-629) conjugated with a highly biocompatible liposomal nanocarrier was shown to target CTSB-overexpressing tumor and stromal cells in the tumor microenvironment, resulting in irreversible and selective inactivation of CTSB, achieving CTSB-specific therapy in cancer (70). DARPins (designed ankyrin repeat proteins) have been shown to block CTSB activity in tumors and have been successfully applied for optical imaging in cancer models. CTSB-selective DARPins represent an attractive theoretical basis for non-invasive diagnostic imaging (71). Although several studies have demonstrated the applications in biochemistry and bioengineering, development of clinical applications such as CTSB-specific inhibitor or antibody still has a long way to go. For instance, the biosafety aspects including the interaction with other drugs, damage to non-targeted organs, drug tolerance, and other related issues need to be further explored by researchers and clinicians.

## Limitations

In present study, we performed a quantitative analysis of the CTSB-related literature using bibliometric methodology. The findings and suggestions may help researchers and clinicians understand the performance and trends of CTSB-related research globally. Nevertheless, due to the inherent limitations of bibliometric approach, we did not examine individual article records to verify the accuracy of indexing. Additionally, we only retrieved publications in a single database and did not search other databases, such as PubMed, Embase, Cochrane Library, etc. This may have led to exclusion of some articles. Furthermore, studies published in earlier years are likely to have higher citations over time, while recently-published studies, even if high-quality, may not have yet acquired comparable visibility. This may have introduced an element of bias and undermined the significance of more recently-published articles. Therefore, researchers should keep abreast of the latest published achievements. Despite these limitations, this research provides a solid global perspective on CTSB-related research over the past decade and highlights the future research direction.

## Conclusion

This bibliometric analysis provides a quantitative synthesis of the published achievements related to CTSB that are available in the Web of Science database during the period 2011 to 2021. Research results and recommendations indicate that USA ranks first in terms of productivity, and the research was performed in close cooperation with other countries, such as China, Germany, England, and Japan. The research output of a country showed positive correlation with GDP. Reinheckel and Sloane BF may be the critical researchers in the field of CTSB. “Plos ONE” was the most productive and cited journal. Analysis of the top citations, co-citations literatures, and the keywords by network map and burst detection clarified the theoretical basis and the key research topics. The hotspots identified in this study included CTSB-related cancer and inflammatory diseases, CTSB-associated cell death pattern, and the applications of CTSB. The topic of CTSB is worthy of continued follow-up by researchers.

## Data Availability Statement

The original contributions presented in the study are included in the article/Supplementary Material, further inquiries can be directed to the corresponding authors.

## Author Contributions

HH and ZL designed and supervised this study. XY, HY, and DZ performed the search. LP and KL collected data. DZ, FC, and CX rechecked data. XY and HY performed analysis. XY and LP wrote the manuscript. All authors read and approved the final manuscript.

## Funding

Funded by the National Natural Science Foundation of China (to HH), No. 81770642, China and research on demonstration application of collaborative network construction in clinical medical research, No. 2015BAI13B08, China.

## Conflict of Interest

The authors declare that the research was conducted in the absence of any commercial or financial relationships that could be construed as a potential conflict of interest.

## Publisher's Note

All claims expressed in this article are solely those of the authors and do not necessarily represent those of their affiliated organizations, or those of the publisher, the editors and the reviewers. Any product that may be evaluated in this article, or claim that may be made by its manufacturer, is not guaranteed or endorsed by the publisher.
